# Spo11: from topoisomerase VI to meiotic recombination initiator

**DOI:** 10.1042/BST20253019

**Published:** 2025-04-02

**Authors:** Jon A. Harper, George G. B. Brown, Matthew J. Neale

**Affiliations:** Genome Damage and Stability Centre, School of Life Sciences, University of Sussex, Brighton, U.K.

**Keywords:** evolutionary biology, meiosis, protein structure, Spo11, topoisomerases

## Abstract

Meiotic recombination is required to break up gene linkage and facilitate faithful chromosome segregation during gamete formation. By inducing DNA double-strand breaks, Spo11, a protein that is conserved in all meiotic organisms, initiates the process of recombination. Here, we chart the evolutionary history of Spo11 and compare the protein to its ancestors. Evolving from the A subunit of archaeal topoisomerase VI (Topo VI), a heterotetrameric type II topoisomerase, Spo11 appears to have evolved alongside meiosis and been present in the last eukaryotic common ancestor. There are many differences between Spo11 and TopVIA, particularly in regulation, despite similarities in structure and mechanism of action. Critical to its function as an inducer of recombination, Spo11 has an apparently amputated activity that, unlike topoisomerases, does not re-seal the DNA breaks it creates. We discuss how and why Spo11 has taken its path down the tree of life, considering its regulation and its roles compared with those of its progenitor Topo VI, in both meiotic and non-meiotic species. We find some commonality between different forms and orthologs of Spo11 in different species and touch upon how recent biochemical advances are beginning to finally unlock the molecular secrets hidden within this fundamental yet enigmatic protein.

## Introduction

Meiosis is of great importance in the evolution of genetic diversity. Recombination, a key feature of meiosis in which homologous chromosomes exchange genetic information, breaks up linkage disequilibrium between genes, allowing new mutations to move to fixation or elimination without the influence of proximal genetic variants [1]. In this way, meiosis allows novel combinations of alleles to arise that would otherwise not be possible or that would take much longer to segregate [2].

While meiosis is central to evolution at the scale of the organism and species, meiosis is fundamentally a cell-biological process that happens within individuals during gamete formation. As such, meiotic defects are highly relevant in the clinical setting, where errors in meiotic processes rank among the most common causes of infertility, particularly in oocytes [3,4]. Even when fertility is not affected directly, chromosomal nondisjunction, in which paired chromosomes or chromatids fail to separate during meiosis, is also the cause of genetic disorders in humans, including not just the well-known Down syndrome but also other trisomies (such as Edwards syndrome, Patau syndrome, and sex chromosome disorders [5–8]), conditions in which an extra copy of a chromosome is present [9], along with conditions in which a chromosome is lost (such as Turner syndrome [10]). It is, therefore, unsurprising that meiosis is the subject of extensive study in many scientific disciplines.

**Figure 1:**
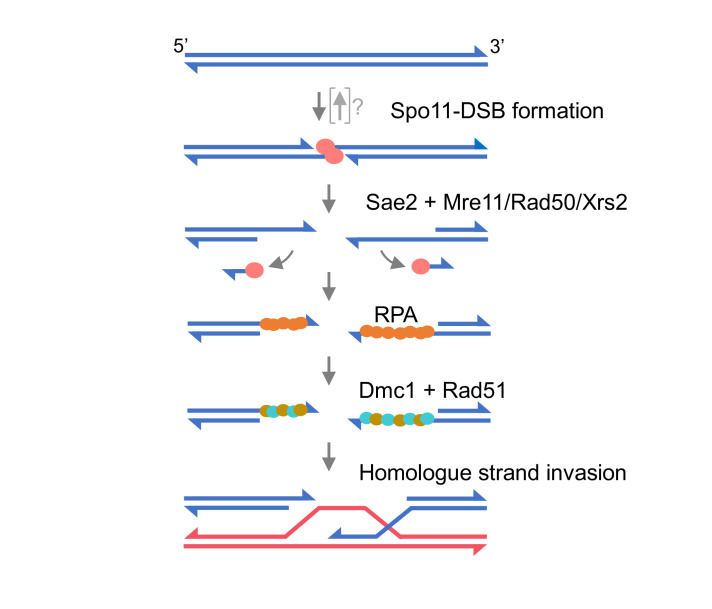
Homologous recombination pathway in meiosis. Homologous recombination pathways during meiosis. The recombination model shows two double-stranded DNA molecules from homologous chromosomes in blue and red, respectively. Spo11 generates DNA double-strand breaks, becoming covalently attached to the 5′ ends, in a topoisomerase-like transesterase reaction. Resection is initiated by the Sae2-MRX (*S. cerevisiae*) or CtIP-MRN in mammalian cells. Single-stranded DNA is coated by Replication Protein A (RPA) and then exchanged for Dmc1 and Rad51, which facilitate strand invasion with the homolog initiating the beginning step of genetic recombination.

Among the molecular machinery involved in the process of meiosis, Spo11 stands out as a vital initiator across nearly all eukaryotic organisms [11]. By creating double-strand breaks (DSBs) in chromosomal DNA, Spo11 induces meiotic recombination to happen [12] (Figure 1). The evolution of the Spo11 protein and its function is, therefore, intimately tied to the evolution of meiosis and the origin of sexual reproduction. Here, we discuss the origins of Spo11, its distribution in the tree of life, and how it has diverged into different variants that share similarities in function and purpose. To that end, we will discuss topoisomerases, the proteins from which Spo11 has evolved, and the reasons why topoisomerases themselves evolved. We will then examine the known variants of Spo11, such as duplicated genes and splice variants, with the aim of revealing new information about Spo11’s current direction of evolution and its functions, which themselves are being informed by recent biochemical characterization.

## The origins and classes of topoisomerases

### The evolution of topoisomerases

Resolution of DNA topological problems is a requirement for all complex life, if it is to maintain genome integrity [13,14]. Through its nature as a long molecular polymer bundled into chromosomes, DNA topology is directly influenced by the stresses induced by DNA replication, transcription, and condensation [15–19]. At the same time, the higher-order DNA conformations created by these processes can directly influence the activity of protein–DNA reactions, fundamentally regulating the very processes that create them [15]. Worse, excess DNA coiling and knotting may prevent chromosome segregation during mitosis and meiosis, effectively becoming lethal for a cell [13,17,20,21]. For these reasons among others, proteins that can alter the topology of DNA, known as topoisomerases, are required. Through their own unique mechanisms, topoisomerases can increase or decrease the torsional stress on DNA and thereby reshape it according to the needs of the cell at any present moment.

DNA genomes are widely believed to have evolved from a genomic world based around RNA [22]—a step that enabled more complex life to evolve via the expansion of the more stable DNA genomes. However, such expansion presents greater challenges to DNA topology. Single-stranded nucleic acid, though less stable, has greater freedom to wind and unwind [23], so such genomes are likely more capable of surviving without substantial topoisomerase activity [24]. By contrast, long double-stranded DNA can more easily coil and knot, building tension and stress that can become lethal [20]. In this context, it is likely that the evolution of topoisomerases enabled the development of larger DNA genomes, thereby facilitating the evolution of more complex life [24–26]. Topoisomerases may, therefore, have evolved from proteins that already operated on DNA, such as DNA polymerases and transposases [24]. Indeed, tyrosine recombinases have been found to have structural and mechanistic homology to one of the many classes of topoisomerase (see below), potentially implicating recombinases as likely ancestors of topoisomerases [27].

An alternative is that DNA topoisomerases evolved from an ancestral RNA topoisomerase. Supporting such a possibility is that a number of topoisomerases of the type IA class possess RNA cleavage activity, such as Topo III [28]. The eukaryotic Topo III probably evolved long after the original topoisomerase; however, RNA activity has also been observed in other type IA topoisomerases from *Escherichia coli* and viruses [29,30]. Because type IA topoisomerases are present across the three domains of life and have RNA activity, it is possible that the first topoisomerase was a type IA topoisomerase that evolved in an RNA genome [31].

### Different classes of topoisomerase

Topoisomerases are split into two types based on their induction of breaks in a single (type I) or both (type II) strands of DNA. By coincidence, type I topoisomerases are classified with odd numbers (Topo I, III, and V) and type II even (Topo II, IV, VI, and VIII). All topoisomerases share a common domain known as TOpoisomerase-PRIMase (TOPRIM)—a ~100 amino acid length domain with two conserved motifs—required to facilitate DNA binding by coordinating divalent metal ions [32–34]. However, the main families of topoisomerases do not share homology, which suggests that they are unrelated and evolved independently [24]. This lack of obvious evolutionary relationships between the topoisomerase classes means that their distributions in different organisms do not necessarily follow phylogenetic trees but instead reflect the environments and needs of the organisms themselves. For example, some unusual topoisomerases such as Topo V have so far been found in only a single species, which is a hyperthermophilic prokaryote living in extreme temperatures with unique challenges to DNA integrity [35]. By contrast, there are also frequent redundancies in topoisomerases in many species—not just from duplicated gene functions such as TOP2 alpha and beta in human cells—but rather also because some topological problems such as DNA stress arising ahead of and behind the elongating transcription machinery can be resolved by both type I and type II topoisomerases [36].

Type I topoisomerases act by transiently cleaving only one strand of DNA. Within this class, type IA enzymes (such as human TOP3) act on partially single-stranded DNA substrates and create strand breaks by linking covalently to the 5´ end of DNA, utilizing a strand passage mechanism [37]. By contrast, type IB enzymes (such as human TOP1) act on duplex DNA and become linked to 3´ ends during the catalytic cycle. Such nicks allow DNA strands to rotate around one another [38–40], creating a ‘swivelase’ activity that can release topological stress that may arise during DNA replication and transcription [36] ; [15–19,41] but are also required for chromosome segregation [13,17,42]. Type II topoisomerases are also split into two families: IIA and IIB, both of which become transiently linked to both 5´ DNA ends that are generated during DNA cleavage due to protein dimerization and coordinated strand-cleavage activity [42,43]. However, one distinction is that type IIA topoisomerases create 4 nucleotide (nt) overhangs, while type IIB topoisomerases produce 2 nt overhangs [44–51].

As discussed in more detail below, Spo11 displays evolutionary and mechanistic similarity to the type IIB family member topoisomerase VI (Topo VI), from which it likely evolved [42]. Spo11, like other type IIB enzymes, also must dimerize to cleave DNA, creating DSBs *in vivo* that have a two-nucleotide 5´ overhang [12,46–50]. However, in contrast with canonical topoisomerases, Spo11 has apparently lost the crucial ability to religate the DSBs that it creates—thus enabling the DNA ends to, instead, be channeled into the recombination pathway that drives genetic variation within sexually reproducing species [46,47,52–56].

## Divergence of Spo11 and its ancestor topoisomerase VI

### Structural relationships between Spo11 and topoisomerase VI

**Figure 2:**
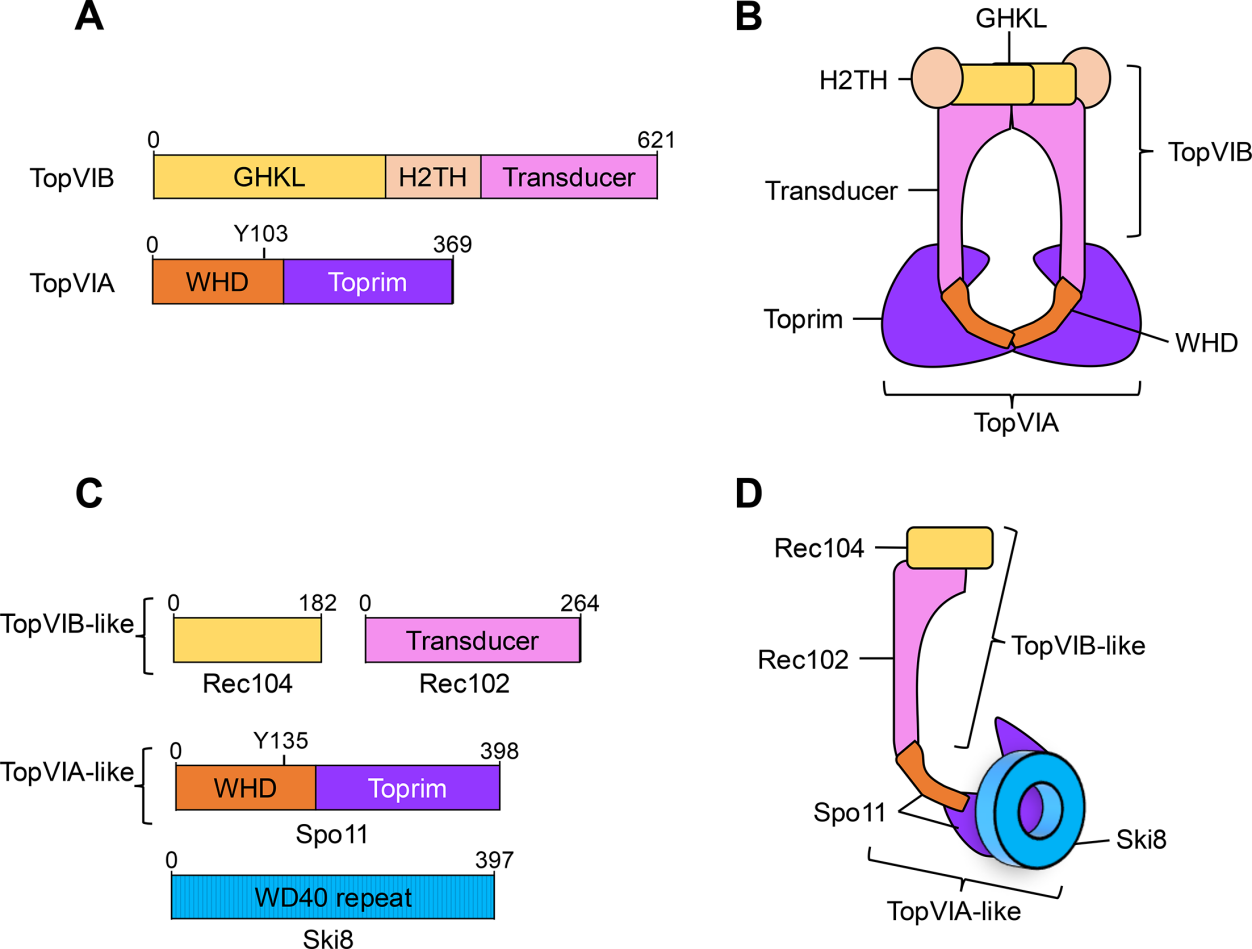
The structure of Topo VI and the Spo11 core complex. **(A**) Domain structure of the A and B subunits of *S. shibatae* topoisomerase VI (Topo VI). (**B**) Cartoon of the Topo VI A_2_B_2_ structure, based on [60]. (**C**) Domain structure of the *S. cerevisiae* Spo11 Core complex: Spo11-Ski8 (TopVIA-like) and Rec104-Rec102 (TopVIB-like). (**D**) Cartoon of the purified Spo11 Core complex (1:1:1:1) structure showing similarity to Topo VI.

Topo VI is a member of the type IIB topoisomerase family [24,42]. First discovered in the archaeon *Sulfolobus shibatae,* Topo VI is thought to be present in nearly all archaea, with the genus *Thermoplasma,* the only exception [57,58]. Topo VI is also present in some bacteria, possibly because of horizontal gene transfer from archaea [59]. Topo VI is a heterotetramer of two subunits: A and B (Figure 2A and B)—a nomenclature that is not to be confused with the A and B designations given to the two families of type II topoisomerases. Although the A subunit of Topo VI appears unrelated to other type II topoisomerases, the B subunit of Topo VI shares similarity with the B subunit of type IIA topoisomerases [38].

**Figure 3:**
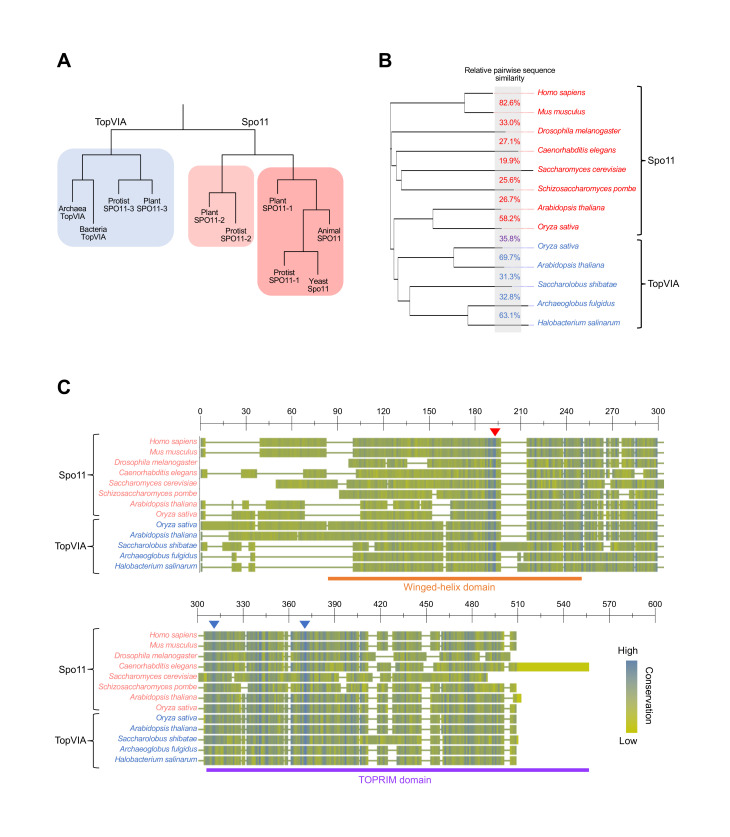
Phylogeny and alignment of Spo11 and TopVIA subunit orthologs. **(A**) Schematic of Spo11 and TopVIA genes, adapted from [64]. (**B**) Phylogeny of Spo11 and TopVIA subunit orthologs in selected species, clustered by sequence similarity. Labels are colored by protein (Spo11 red, TopVIA subunit blue). Numbers between species names indicate the pairwise sequence similarity between proteins in adjacent nodes. (**C**) Protein alignment of Spo11 and TopVIA subunits. Colors represent BLOSUM conservation scores, with blue residues identical between proteins. The orange line indicates the winged helix domain, and the purple line indicates the TOpoisomerase-PRIMase (TOPRIM) domain (see Figure 2). In *S. cerevisiae*, residues 35 to 170 comprise the winged helix domain, and residues 227 to 397 comprise the TOPRIM domain. The red arrowhead indicates the position of the catalytic tyrosine residue, which is conserved in all Spo11 and TopVIA subunit orthologs. Blue arrowheads indicate the positions of conserved magnesium and manganese binding residues. Species names in red represent Spo11, species names in blue represent TopVIA subunit. Alignment was performed using the NCBI COBALT alignment tool (BLOSUM 80, [65]).

Spo11 bears structural similarities to the A subunit of Topo VI [42,61] (Figure 2C and D), from which it is widely believed to have evolved [42,60]. Unusually for a meiosis-specific protein—many of which seem to be subject to relatively rapid change [11,62]—Spo11 has many easily identified orthologs [63], suggesting that an essential function of Spo11 is limiting its ability to undergo substantial evolutionary change. Nevertheless, despite such likely functional conservation, sequence conservation between orthologs in other species is relatively low (between 20% and 30%) (Figure 3A and B) [11,42].

Although Spo11 is found across many species, it was originally believed that a B subunit of Spo11 was absent—perhaps because it was not required for Spo11 function due to Spo11 not acting as a canonical type IIB topoisomerase. However, a protein with homology to the B subunit, named Top6BL (B-like), has subsequently been discovered in mice and Arabidopsis [66,67]. Top6BL has high divergence but also substantial conservation with TopVIB across eukarya [68]. Orthologs of Top6BL are also found in proteins known previously to be essential for Spo11’s catalytic function *in vivo*: Rec102-Rec104 in *S. cerevisiae* and Mei-P22 in *D. melanogaster* [61,67]. The evolutionary connection between these proteins and the ancestral Top6B subunit likely evaded prior detection because of very low sequence identity: around 11% between orthologs [67]. Furthermore, Top6BL and its orthologs notably differ from Top6B in the lack of a helix-2-turn-helix domain (Figure 2) [64], reducing sequence and structural similarity.

Collectively, Spo11 and Top6BL are now considered to form the functional holocomplex relevant to the formation of the DSBs that initiate recombination in meiosis via a modified topoisomerase-like mechanism [61,69]. In *S. cerevisiae*, alongside Spo11 and Rec102-Rec104, this ‘core complex’ includes the Ski8 protein—seemingly co-opted as a scaffold protein otherwise involved in RNA metabolism [61,70] (Figure 2C and D). Such functional interactions corroborate known genetic dependencies and detailed yeast two-hybrid interaction data [70,71]. Thus, this core complex is now thought to be required for Spo11 to function, just as Topo VI requires both its A and B subunits to function [72]. As such, even though there are evolutionary differences, both Top6BL and Spo11 are, therefore, conserved across meiotic organisms [11,42,67,68].

Both TopVIA and Spo11 are composed of two domains. Alongside the TOPRIM domain, TopVIA and Spo11 contain a winged-helix domain, both of which interact with DNA [32,61,73]. One of the residues in the winged-helix domain is a particularly important catalytic tyrosine that is required for the formation of DSBs and conserved in all variants of TopVIA and Spo11 [74] (Figure 3C). Two metal binding residues are also conserved in all variants of TopVIA and Spo11, and like in other transesterases, they are critical for DSB formation (Figure 3C) [69,73].

### Mechanistic differences between Spo11 and Topo VI

**Figure 4:**
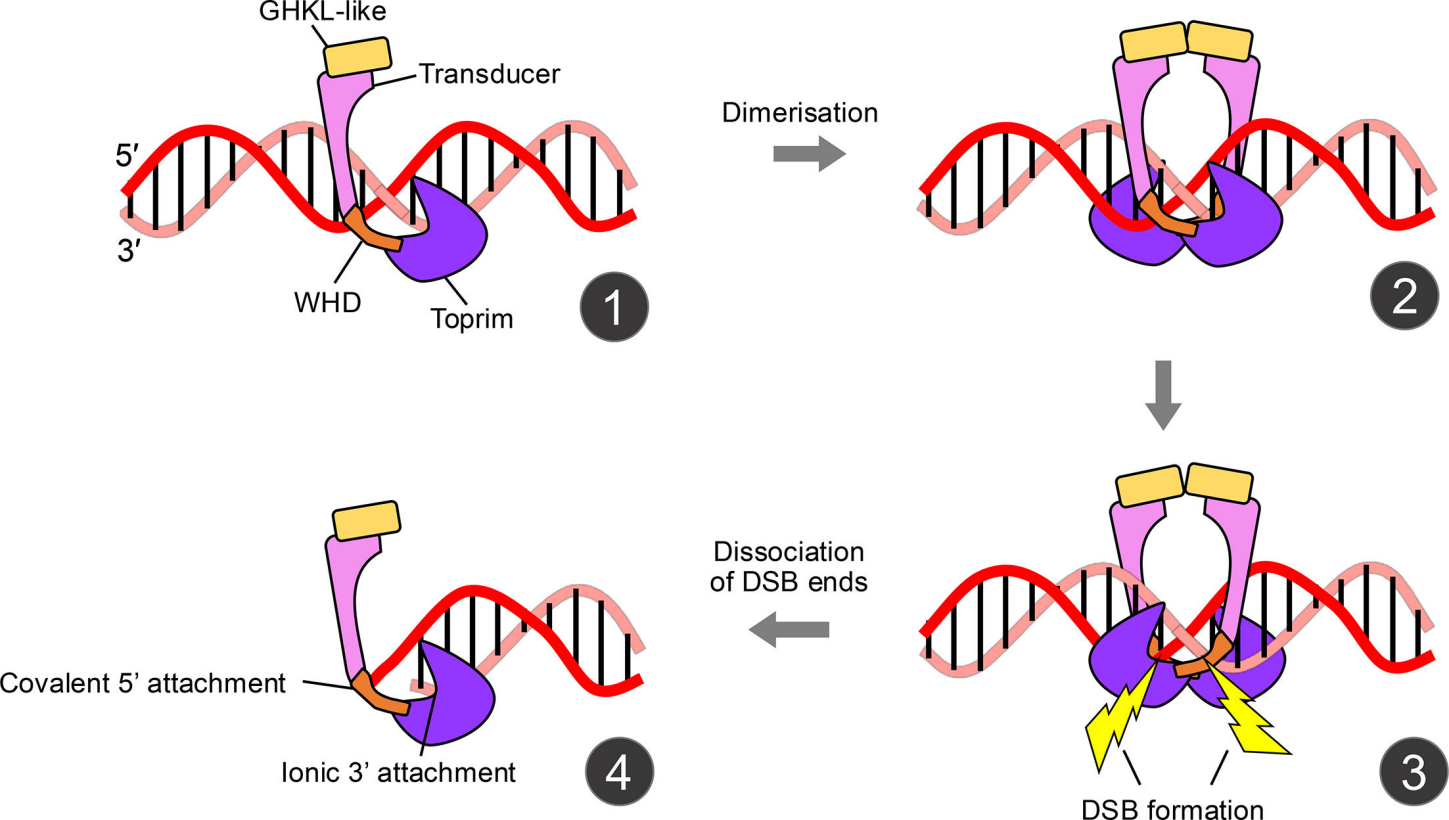
Mechanism of Spo11 action. A schematic of how the Spo11 core complex may interact with the DNA and generate double-strand breaks (DSBs). (**1)** The Spo11 core complex binds DNA ionically as a monomer. (**2)** A second Spo11 core complex is recruited—promoted by high local protein concentration, and likely mediated by additional pro-DSB factors ‘RMM’ (not drawn). (**3)** Spo11-DSB catalysis occurs, leaving Spo11 covalently linked to the two 5′ ends of the DNA and creating 2 nt overhangs. (**4)** Dissociation of DSB ends from one another leaves the Spo11 core complex bound to the 5′ strand covalently and the 3′ ends ionically, potentially clamping both the 5′ and 3′ strands together and preventing the end degradation.

Such structural similarity between Topo VI and Spo11 suggests a shared mechanism for DNA cleavage. Topo VI has been proposed to use a two-gate strand-passage mechanism similar to that employed by type IIA topoisomerases such as Topo II [75]. The two-gate model involves a covalent linkage between the dimerized type II topoisomerase and double-stranded DNA via the catalytic tyrosine residue present within the winged-helix domain, with this and the TOPRIM domains forming a gate with the GHKL and transducer domains (Figures 2 and 4, [60]). Strand passage itself—a staple of topoisomerases, yet a reaction believed impossible to be catalyzed by the Spo11 core complex—requires the capture of a second segment of DNA in a process requiring the presence of ATP prior to DNA cleavage [44,76]. The ATP binding domain in TopVIB is the GHKL domain, which can also be found in a handful of other protein families such as Hsp90, DNA mismatch repair enzymes (MutL), and histidine kinases [42,75,77–79]. Once the DNA is broken, the hydrolysis of ATP allows the second segment to pass through the DSB gap [75].

With this in mind, it is conspicuous that the Spo11 core complex has no identifiable ATPase domain, unlike Topo VI [61,66,67,80]. Such absence hints that the essential role that ATP binding and/or hydrolysis plays in the catalytic DNA cleavage cycle of canonical Topo VI topoisomerases is no longer relevant or essential for Spo11 to initiate recombination. Moreover, the lack of ATP binding/hydrolysis may have caused Spo11 to become a mechanistically amputated topoisomerase—evolutionarily selected specifically for the job of inducing DSBs that then require repair by recombination. Indeed, ATP hydrolysis has been suggested to prevent the potentially toxic generation of DSBs, whereas—by contrast—DSB formation is essential for meiotic recombination [81].

Notably, while Topo VI can act on both over- and underwound DNA substrates [24,57], in the absence of ATP hydrolysis, Topo VI appears to preferentially bind and cleave underwound DNA [82]. Consistent with the lack of an identifiable ATPase domain in Top6BL, neither ATP binding nor hydrolysis is required for Spo11 cleavage [83–85], unlike Topo VI which requires ATP binding but not hydrolysis [60,82,85]. Therefore, the lack of ATP dependence may have contributed to a situation where the Spo11 core complex preferentially acts on underwound DNA. Indeed, *in vivo*, Spo11 initiates recombination by preferentially forming DSBs in nucleosome-free regions of the genome, such as gene promoters, where DNA is both accessible and predicted to be underwound [48,50,86,87]. Moreover, recent studies, *in vitro* and *in silico,* have further confirmed Spo11’s preference for bendable and underwound DNA [83,84].

## Contrasting regulation of Spo11 and Topo VI

### Spo11 is subject to stricter control than topoisomerases

Although Spo11 is closely related to type II topoisomerases, Spo11 appears to be much more tightly regulated. Spo11 is only expressed early in meiosis in both mice and *S. cerevisiae* [88,89]. Furthermore, Spo11 is widely believed to be subject to suicide inhibition—after catalyzing the formation of a DSB and undergoing cleavage and endonucleolytic release, Spo11 remains bound to a short DNA oligonucleotide, thus becoming unable to catalyze another DSB [52,56,90–92]. However, recent biochemical evidence has emerged that suggests Spo11 nonetheless has the capacity to reseal single-stranded breaks should it be coerced into creating them [83,84]. Thus, such activity suggests it is possible that Spo11 can, in principle, do the same with DSBs—an activity that has long been considered [52].

Unlike topoisomerases, Spo11 requires numerous additional proteins to function *in vivo*. For example, in *S. cerevisiae*, the organism in which the mechanism of Spo11 was originally discovered [42,52], nine proteins are required for Spo11 to act: the aforementioned core complex members (comprising Rec102, Rec104, and Ski8; Figure 1), Rec114, Mer2, Mei4 (‘RMM’), and the MRX complex (consisting of Mre11, Rad50, and Xrs2) [11,61]. Additionally, Spo11 activity is strongly influenced by cohesin [93,94] and proteins of the HORMAD family such as *S. cerevisiae* Hop1, which is capable of directing Spo11 toward specific genomic sites [95,96]. Furthermore, Spo11 seems to act more than would otherwise be expected on smaller chromosomes, a phenomenon that depends on pro-DSB factors such as Hop1 [48,96–98]. Orthologs of Hop1 are present in mammals, flies, worms, and plant species, highlighting functional conservation in mechanism and/or regulation [99]. In mammals, Spo11 activity is further spatially modulated by the sequence-specific histone methylating activity of PRDM9, a rapidly evolving gene linked to speciation [100–103].

### Regulation of Spo11 activity

How exactly Spo11 activity is regulated remains of great interest. Accumulating evidence supports the view that Spo11-DSB formation is regulated both proactively (by those pro-DSB factors mentioned above) and reactively (by downstream of DSB formation) [104]. This latter layer of regulation can exist because of the asynchronous generation of numerous DSBs across the genome in each meiotic prophase. Such spatial and temporal separation creates a system in which the likelihood of forming any subsequent DSB can be influenced by earlier DSBs [105,106]—a process termed DSB interference [86,107,108]. Nevertheless, the precise targets of such regulation remain undetermined—although the pro-DSB factors remain the most likely candidates [97,107,109–112].

Due to the relative simplicity of topoisomerase regulation—and a potential ubiquitous requirement for their activity—there is yet no evidence to suggest that topoisomerases are subject to this same level of control. Nevertheless, topoisomerase activity may indirectly be subject to regulation due to the nature of topoisomerase catalytic action being more likely in areas of higher torsional stress [41,113]. Specifically, localized pockets of topological stress might lead to concerted activity (a feature also common to Spo11 [86,87,107]); also yet, such stresses, when released by topoisomerase action, may indirectly act to suppress the requirement for further activity in the vicinity (with parallels to Spo11-DSB interference). While it is known that the DNA damage response kinase ATM (Tel1 in *S. cerevisiae*) is required for Spo11-DSB interference [105,107,108], whether such a mechanism also requires and/or is mediated by changes in local DNA topology and/or stress is not known.

### Spo11 is only active when dimerized

The Spo11 core complex, much like other type II topoisomerases, is only catalytically active when dimerized [46–50]. *S. cerevisiae* Spo11 is only stable in solution as a core-complex monomer and not as a dimer [61]—something that also holds true for the mouse proteins [83–85]. It is, therefore, possible that the Spo11 core complex binds DNA as a monomer but is only catalytically active upon (potentially transient) dimerization. Further, it was proposed that Spo11 may act on the surface of phase-separated condensates of pro-DSB factors (such as Hop1, Rec114-Mei4, and Mer2) [86,87,110]. Such condensates may act as a mechanism to increase the local concentration of Spo11 above a critical point, thereby promoting dimerization and subsequent cleavage. Consistent with this view, *in vitro* DNA cleavage by Spo11—recently demonstrated by purified mouse Spo11 [83–85]—is strongly stimulated by high protein-to-substrate concentration [83,85] (Figure 4).

It is interesting to consider that the complex regulation of Spo11 may have evolved—unlike for topoisomerases—to down-regulate what could otherwise become highly toxic genome-destabilizing DNA breaks. Nevertheless, the restriction of Spo11 activity to within regions of high local concentration appears to come with its own dangers, including the formation of hyper-localized coincident DSBs (termed double-cuts or double-DSBs [86,87]) that can lead to gap repair and/or complex insertions and rearrangements [114].

## Meiotic and non-meiotic roles of modern Spo11 variants

### Conservation of Spo11 function

**Figure 5:**
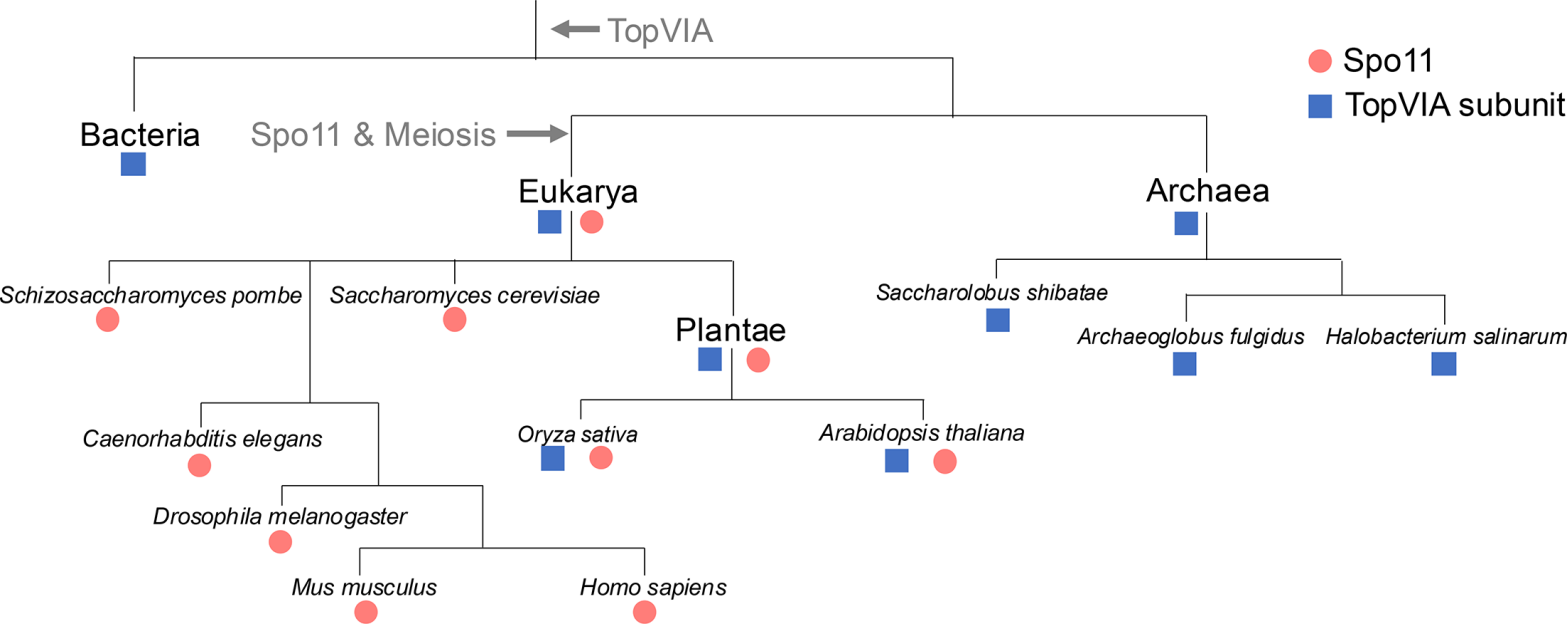
Phylogeny of Spo11 and TopVIA subunits. Spo11 can be found in the genomes of species marked with a red circle. TopVIA subunits can be found in the genomes of species marked with a blue square.

Spo11 is only known to be present in eukarya, where its ubiquitous presence suggests that it was already present in the last common eukaryotic ancestor, evolving from TopVIA in a prior period of time (Figure 5). The distribution of Spo11 in the tree of life is logical, considering its functionality in meiosis. When comparing two unrelated organisms where both Spo11 and TopVIA are present, Spo11 and TopVIA are more similar to their orthologs than they are to each other (Figure 3B), suggesting that Spo11 evolved once and supporting the theory that Spo11 could be found in the last common eukaryotic ancestor. Nevertheless, the exact timing of Spo11 emergence is difficult to discern, given the evolutionary timescales involved. However, given that the generation of DSBs in meiosis is the only well-attested conserved function of Spo11 [115], it is likely that Spo11 and meiosis originated at around the same time.

Despite the apparent importance of Spo11 to meiosis, there are examples of sexually reproducing species that can produce gametes in its absence. For example, the lack of recombination in male *Drosophila melanogaster* means they can undergo meiosis without the Spo11 homolog *mei-W68* [116], and other insect species have been reported to be capable of the same [117,118]. It is important to note, however, that female *D. melanogaster* do undergo recombination and require *mei-W68* to do so*—*which is expressed in oocytes—likely explaining the retention of Spo11 in this species even when it has no role in male meiosis [116].

Although Spo11 has a vital, well-conserved function, there is evidence that it may have taken on additional non-meiotic roles in some species. Chief among these is the fact that Spo11 orthologs are found in some asexual species such as Giardia, *Candida albicans,* and *Acanthamoeba castellanii*, which precludes any meiotic role [62,119,120]. Notably, Spo11 is conserved within these species, even after it would be expected to degenerate if not playing an active role, and after genes coding for other essential meiotic functions such as the kinase, inducer of meiosis has been lost [121]. Such findings suggest evolutionary selection for a functional Spo11 gene outside of its archetypal meiotic role.

### Spo11 splice variants

Long after the evolution of meiosis, new variants of Spo11 have continued to arise, potentially to provide new and more diverse functions and/or regulation. For example, mammals possess two splice variants of Spo11: α and β. The β isoform contains exon 2, whereas the α isoform skips it [122]. Interestingly, early-stage meiotic spermatocytes primarily synthesize Spo11β, with Spo11α synthesized more in late meiosis [88,123], where it appears to play an important role in facilitating recombination between the short pseudoautosomal regions (PAR) present on the XY chromosome pair [124], perhaps due to the PARs unique structural organization [124,125]. Consistent with this sexually dimorphic role for Spo11α, female mice expressing only Spo11β are fully fertile, while male mice are not [124].

Differential splicing of Spo11 also arises in plants such as *Arabidopsis thaliana, Oryza sativa, Brassica rapa,* and *Carica papaya* [126], where as many as seven different splice variants of Spo11 have been identified [127]. How the functions of these different splice variants differ is not yet known, so it remains unclear why such variation evolved. However, most appear to generate non-functional truncated protein [126]. Interestingly, plant splice variants tend to vary by the retention or exclusion of introns, rather than exons, contrasting mammalian splice variants and potentially indicating slight functional variation between plant and animal Spo11 [122,126].

### Spo11 paralogs

In addition to splice variants, at least three different homologous Spo11 proteins have been found in plants [72]. These proteins, known as SPO11-1, SPO11-2, and SPO11-3, exhibit low sequence similarity and are likely ancient paralogs rather than the result of recent duplication [128]. All three homologs share the catalytically active tyrosine residue (position 103 in *A. thaliana* SPO11-1 protein), which is essential for the generation of DSBs [42,74,129].

While Arabidopsis SPO11-1 and SPO11-2, like Spo11 in other organisms, are essential for DSB formation in meiosis [128], specific roles for these two homologs remain unclear. SPO11-1 and SPO11-2 are believed to form a complex with each other and Top6BL, together performing the same role as the Spo11 core complex in other species [66]. Interestingly, the two Spo11 proteins appear to act in the same pathway as each other, for knocking out both results in the same phenotype as only knocking out one [74,128]. However, whether this indicates a requirement for heterodimerization, or—similar to in mouse alpha and beta splice variants—an essential, yet independent role for each paralog, remains unclear. The recent demonstration of catalytic activity for the mouse proteins *in vitro* opens the door for potentially testing the capacity and functionality of heterodimerization of Spo11 from other species. Indeed, it is interesting to consider that, once duplicated, the two Spo11 paralogs evolved co-operatively to take one position each and form an obligate heterodimer [11]. Yet, even if so, why such a dependency between paralogs might evolve remains unclear.

The third Spo11 variant in plants, SPO11-3, was initially identified and named due to its similarity to Spo11 [72]. However, it is now widely believed that SPO11-3 actually encodes the A subunit of the plant Topo VI protein, due in part to its lack of a meiotic function [128,130–133]. Interestingly, unlike in mammals, the conservation of both SPO11-1/-2 and TopVIA (SPO11-3) functions suggests that both meiotic and non-meiotic pathways retain critical roles for cell and/or species viability in plants. With this in mind, the TopVIA protein encoded by SPO11-3 has a role in somatic endoreduplication, an alternative cell cycle process in which a cell replicates its genome without dividing [134]. This process occurs only in certain tissues in mammals, such as megakaryocytes in mice for platelet formation [135]. However, in plants, this process is relatively widespread among the cell tissue types and is thus vital for vegetative growth [131,136]. Interestingly, although SPO11-1/-2 and SPO11-3 appear to display different functions, they share greater sequence similarity (~35%) in *A. thaliana* (Figure 3A and B) than that observed when comparing TopVIA orthologs between species (25–30% sequence similarity, Figure 3A and B).

In *O. sativa*, two further Spo11 homologs have been discovered*,* which seem to be only present in this rice species [137]. One of them, OsSpo11-4, was believed to be essential for meiosis [137]. However, later evidence instead suggested that it does not have a meiotic role [138]. The other, OsSpo11-5, also appears to have no role in meiosis, and its function has yet to be described [138]. For both, the close functional and evolutionary similarity of Spo11 and TopVIA subunits suggests that, like SPO11-3 in Arabidopsis, these proteins may actually be subunits of Topo VI and thus involved in other aspects of genome maintenance.

## Discussion

**Figure 6:**
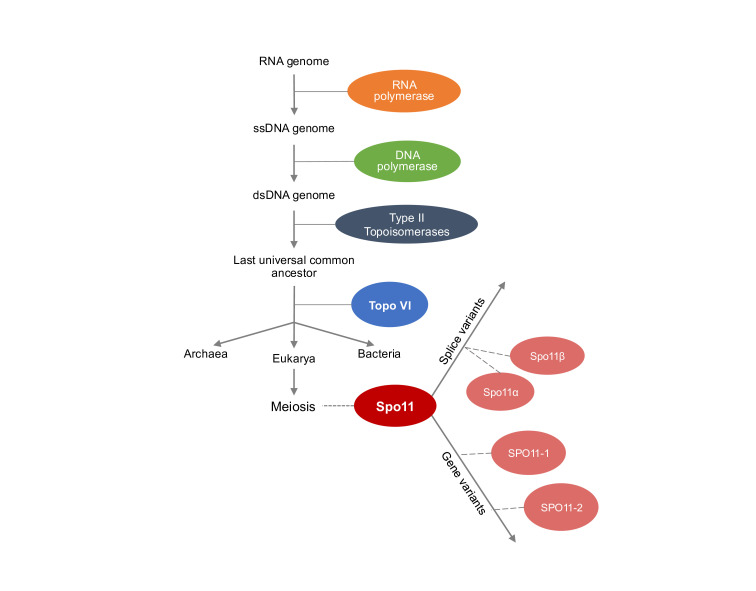
Evolutionary history of Spo11. A schematic of key events in the evolution of Spo11. Proteins in the ancestry of Spo11 are connected to the timeline at the point where they are believed to have originated.

Anywhere meiosis exists, Spo11 can also be found. It is an ancient and effective protein that has functionally changed relatively little throughout evolutionary history, a testament to the fundamental importance of its function. Nevertheless, variants of Spo11 appear to have evolved and been lost over time (Figures 5 and 6). Yet, in addition, a common thread between animals and plants is the facilitation of one variant by another—perhaps aiding their retention. For example, from the more direct interaction between variants observed in plants, to the complementary effects of isoforms found in mice, both seem to suggest that two Spo11 variants can be better than one alone. If that is the case, then why are some variants lost? It could be that a second variant imparts only minor or slight benefits, which is supported by the relatively mild phenotypes observed when only one is removed. Perhaps, a second Spo11 is only beneficial under certain conditions and for specific life histories. If so, then the appearance and phylogenetic distribution of Spo11 variants is not unlike that of the topoisomerase families themselves.

Despite the initial detection of DSBs as the initiators of meiotic recombination some 35 years ago [139], and the identification of Spo11 as the catalytic entity less than a decade later [42,52], there have, nonetheless, remained major challenges to understanding Spo11 in greater mechanistic detail. The recent, groundbreaking demonstration of robust catalytic activity of the mouse protein *in vitro* [83–85], alongside the detailed understanding of the structural organization of protein interactions within the core complex [61], is likely to usher in a new wave of mechanistic information that will inform and inspire future cellular and molecular investigations. Nonetheless, outside of studies in unicellular organisms, *in vivo* cellular work in multicellular species will likely remain a challenge for some time. Investigations in spermatocytes are difficult, but female meiosis proves to be truly challenging, where oocyte numbers are inherently limited [140]. As a result, without a suitable cell culture model—and even with one—we may only ever see half the picture of Spo11’s function and regulation in more complex life such as mammals, severely limiting any study of sex-specific differences.

Years after its discovery, there is still much we do not know about Spo11. The relatively recent discovery of Top6BL has challenged much of what had been deduced with regard to the requirements of Spo11 to function and its mistaken lone propagation throughout eukaryotic species [66,67]. New variants and homologs of Spo11 continue to be discovered, and each one reveals either a more nuanced, situational utility of Spo11 in meiosis or an entirely new function altogether. Wherever the next discovery is to arise, it remains clear that the key to understanding the core, vital components of meiosis, and its evolution may lie within this remarkable protein.

**Perspectives**

- Spo11 is the highly conserved key initiator of meiotic recombination, vital for the generation of genetic diversity. The study of Spo11 is important not just to the understanding of chromosome mis-segregation disorders linked to meiosis but also to genome stability.
- Spo11 is widely believed to have evolved from topoisomerase VI (Topo VI), an archaeal enzyme, and was likely present in the last common eukaryotic ancestor. Spo11 is also subject to extensive regulation, beyond that experienced by Topo VI, and variants have evolved to refine its function.
- Recent *in vitro* discoveries elucidate core features of the mechanism of Spo11 action such as the requirement for dimerization and have revealed conservation between the B subunit of Topo VI and the Spo11 core complex. Our understanding of Spo11 action and its requirements may soon be transformed.

## Abbreviations

DSBs: double-strand breaks
PARs: pseudoautosomal regions
TOPRIM: TOpoisomerase-PRIMase
Topo VI: topoisomerase VI
